# Management of Obesity and Obesity-Related Disorders: From Stem Cells and Epigenetics to Its Treatment

**DOI:** 10.3390/ijms24032310

**Published:** 2023-01-24

**Authors:** Sara Cruciani, Alessandro Palmerio Delitala, Maria Laura Cossu, Carlo Ventura, Margherita Maioli

**Affiliations:** 1 Department of Biomedical Sciences, University of Sassari, 07100 Sassari, Italy; 2 Consorzio Interuniversitario “Istituto Nazionale Biostrutture e Biosistemi” (INBB), Viale delle Medaglie d’Oro 305, 00136 Roma, Italy; 3 Department of Medicine, Surgery and Pharmacy, University of Sassari, 07100 Sassari, Italy; 4 General Surgery Unit 2 “Clinica Chirurgica” Medical, Surgical and Experimental Sciences Department, University of Sassari, 07100 Sassari, Italy; 5 National Laboratory of Molecular Biology and Stem Cell Engineering, Eldor Lab, Istituto Nazionale di Biostrutture e Biosistemi (INBB), Via di Corticella 183, 40128 Bologna, Italy; 6 Center for Developmental Biology and Reprogramming (CEDEBIOR), Department of Biomedical Sciences, University of Sassari, Viale San Pietro 43/B, 07100 Sassari, Italy

**Keywords:** adipose-derived stem cells, epigenetics, adipogenesis, obesity, cellular mechanisms, bariatric surgery, anti-obesity drugs

## Abstract

Obesity is a complex worldwide disease, characterized by an abnormal or excessive fat accumulation. The onset of this pathology is generally linked to a complex network of interactions among genetic and environmental factors, aging, lifestyle, and diets. During adipogenesis, several regulatory mechanisms and transcription factors are involved. As fat cells grow, adipose tissue becomes increasingly large and dysfunctional, losing its endocrine function, secreting pro-inflammatory cytokines, and recruiting infiltrating macrophages. This long-term low-grade systemic inflammation results in insulin resistance in peripheral tissues. In this review we describe the main mechanisms involved in adipogenesis, from a physiological condition to obesity. Current therapeutic strategies for the management of obesity and the related metabolic syndrome are also reported.

## 1. Introduction

Lifestyle changes, such as the consumption of calorie-dense foods and sedentary living, have progressively led to a profound imbalance between calorie intake and consumption [1]. Obesity is a direct consequence of these changes and is closely related to many metabolic disorders, including type 2 diabetes, insulin resistance, hyperglycemia, dyslipidemia, hypertension, and non-metabolic diseases, such as heart disease and many types of cancer [2,3,4]. The prevalence of childhood/adolescent obesity is increasing worldwide, rising from 4.5 percent in 1990 to 6.7 percent in 2010 [5]. A survey of the obesity epidemic shows that as of 2022 more than 39 million children worldwide are obese [6]. Obesity is strictly dependent on body composition rather than body weight and particularly on the number of adipocytes. Adipose tissue can be classified into different types, white (WAT) and brown (BAT) [7]. The primary function of WAT is energy storage in the form of triglycerides (TG) [8]. On the other hand, BAT dissipates energy to produce heat, suggesting its possible anti-obesity role [9,10]. In addition to brown and white adipocytes, another class of adipocytes, called beige/brown adipocytes, has recently been described [11]. Preadipocytes are converted into mature adipocytes in the final stage of differentiation when they are exposed to certain stimuli [12]. Thus, identifying the molecular mechanisms underlying resident mesenchymal stem cell (MSCs) differentiation could add new insights for the identification of future therapeutic approaches against obesity [13]. Mesenchymal stem cells (MSCs) are a type of adult stem cells known for their high plasticity and ability to generate mesodermal and non-mesodermal tissues [14]. Cell proliferation and differentiation are two opposing processes. Adipogenesis is regulated by a complex and highly orchestrated gene expression program, which occurs in stages [15]. The first phase involves the commitment of pluripotent stem cells into preadipocytes. Preadipocytes cannot be morphologically distinguished from their precursor cells, but they have also lost the potential to differentiate into other cell types [16]. Therefore, in the second stage, known as terminal differentiation, preadipocytes gradually acquire the features of mature adipocytes and become able to regulate lipid transport and synthesis, insulin sensitivity, and secretion of adipocyte-specific proteins [17]. During these phases, specific adipogenic-related genes are induced, and, at the same time, a series of epigenetic and chromatin modifications also occurs, leading ultimately to the silencing of the stemness-related genes [18,19]. Several natural or synthetic molecules can therefore act directly on these transcription factors, thus representing promising therapeutic agents to modulate uncontrolled adipose tissue hyperproliferation [20,21]. In this review, we focus on the biological features of adipose tissue, analyzing the transcription factors and key proteins involved in stem cell differentiation and the major surgical and pharmacological interventions for the control of obesity and its related metabolic syndrome. 

## 2. Epigenetic Programming of Adipose-Derived Stem Cells (ADSCs)

**Figure 1:**
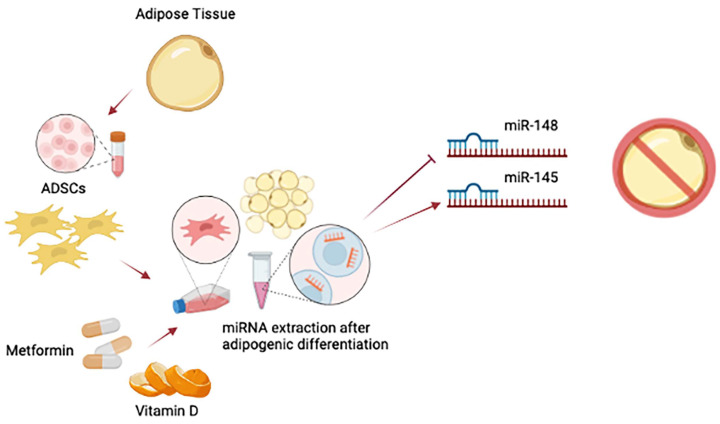
Interaction between molecules and miRNAs. ADSC exposure to metformin and vitamin D modulate miRNAs profile, inducing the upregulation of miR-145 and the simultaneously downregulation of miR-148. The final effect is the inhibition of adipogenesis. Created with BioRender.com accessed on 30 December 2022.

Adipose-derived stem cells (ADSCs) have emerged in recent years as the most sought-after source of cells for the treatment of obesity, metabolic and degenerative diseases, due to their availability, rapid expansion, and differentiative potential toward several phenotypes [22,23]. ADSCs are mainly found in the so-called stromal vascular fraction (SVF) of adipose tissue, from which they can be easily isolated by mechanical and enzymatic procedures [24,25]. The application of adult stem cells in regenerative medicine enables the repair of many tissues, including vessels, muscles, nerves, cartilage, and skin [26]. Stem cell differentiation requires the precise activation of genes involved in the development of a definite cell type. On the other hand, differentiation itself involves the suppression of specific stemness-related genes, such as octamer-binding transcription factor 4 (Oct-4), sex determining region Y-box 2 (SOX2), and Nanog Homeobox protein (NANOG) [27,28]. The differentiation of preadipocytes into mature adipocytes is regulated by a complex network of transcription factors [29,30]. Any dysregulation in this process causes lipodystrophy, which impairs glucose and lipid homeostasis [31]. While Runt-related transcription factor 2 (RUNX2), the key osteogenic transcription factor, triggers an osteogenic differentiation program in MSCs, adipogenic differentiation is mainly promoted by peroxisome proliferator-activated receptor gamma (PPARγ) and CCAAT/enhancer binding protein alpha (C/EBPα) [32,33,34]. PPARγ, in particular, is considered the master regulator of adipogenesis [35]. On the other hand, C/EBPα-deficient cells are able to differentiate as adipocytes; however, these C/EBPα-deficient cells are resistant to insulin [36]. In addition to PPARγ, other genes are also involved in adipogenic commitment, such as uncoupling protein 1 (UCP1), which distinguishes BAT from WAT, whose function is thermogenesis in response to cold stress or β-adrenergic stimulus [37]. Insulin-Like Growth Factor-1 (IGF-1), Transforming Growth Factor beta (TGF-β), or cyclic AMP (cAMP) signaling pathways also play key roles in adipocyte differentiation [38]. Moreover, differential gene expression appears to be strongly influenced by epigenetic factors. DNA demethylation and methylation, acetylation or ubiquitylation of histones, as well as various noncoding RNAs, such as microRNAs, are the most studied epigenetic factors involved in modulating cellular organization [29,39]. Epigenetic changes can directly modulate gene promoters, thus facilitating (or preventing) the recruitment of additional chromatin-modifying enzymes or transcriptional regulators that would drive stem cell differentiation [40,41]. Post-translational modifications (PTMs) of histones by histone deacetylases (HDACs) or histone methyltransferases (HMTs) have been reported to be crucial in shaping the process of adipogenesis [42,43]. Reduced expression of Sirt1 and Sirt2, for example, has been associated with increased differentiation capacity of visceral adipose stem cells [44]. In addition, altered global DNA methylation pattern was observed during metabolic disorders on various genes involved in adipocyte differentiation, lipid metabolism, and inflammation [45,46]. Decreased expression of some epigenetic factors, such as HDAC1, promotes adipogenesis and visceral fat accumulation in human obesity [47,48]. Within this context, it has been demonstrated that exposure of ADSCs to metformin and vitamin D increases the expression of HDAC1 and other epigenetic modulators [49]. Furthermore, several microRNAs (miRNAs) are indeed found to be involved in the inactivation of the adipogenesis process. After treatment with metformin and vitamin D, for example, ADSCs showed upregulated levels of miR-145 and a downregulated expression of miR-148 [49]. miR-145 is downregulated during adipogenesis, while its upregulation inhibits adipogenesis by reducing the activity of PI3K/Akt and MAPK signaling pathways [50]. The expression of miR-148a is also affected by lipid accumulation. When upregulated, miR-148a promotes adipogenic differentiation, while it inhibits preadipocyte differentiation when miR-145 is upregulated and miR-148 is downregulated [51] (Figure 1). These molecules are also able to modulate inflammation and the expression of other key genes involved in adipogenic differentiation, counteracting WAT formation, and inducing a “brown-like” phenotype [52,53]. The use of bioactive molecules and chemical stimuli can then control the de-novo lipogenesis, differentiation, and physiology of adipose tissue, for the in vivo treatment of chronic pathological conditions of difficult resolution.

## 3. Biological Features of Adipose Tissue

Adipose tissue comprises 15–25% of the body weight and it can be classified into subcutaneous and visceral tissue [54]. Adipose tissue represents the main site of energy storage playing an important role as an endocrine organ, and directly modulating systemic lipid, glucose metabolism, and insulin sensitivity [55]. Within adipose tissue, in addition to the adipocytes, the stromal vascular fraction (SVF), containing a type of stem cells called adipose-derived stem cells (ADSCs), can be found [56,57]. These cells are able to differentiate into mature adipocytes following a process called adipogenesis [58]. Within healthy adipose stores, ADSCs have extensive immunomodulatory functions, such as the inhibition of natural killer (NK) cell and T cell proliferation. Moreover, they exert key secretory functions by releasing inflammatory cytokines, as interleukin-6 (IL-6) and tumor necrosis factor-alpha (TNF-α) in response to inflammation [59,60]. Lipid storage is determined by the balance between lipogenesis and lipolysis. The volume of the adipocyte reflects its specific function of storing energy in the form of lipids and, therefore, the ability of the cell to drastically modulate its size in response to changes in energy balance [61]. Adipose tissue expansion is determined by hyperplasia and/or hypertrophy of adipocytes. Hyperplasia refers to the formation of new adipocytes from preadipocytes at-hormone regulation and mediated by a series of transcription factors [30]. Unlike hyperplasia, hypertrophy is the enlargement of adipocytes by accumulation of lipids, either by uptake from the circulation or through the fatty acid synthesis pathway, known as “de novo lipogenesis” [62]. Generally, adipocyte hypertrophy is mainly associated with insulin resistance, hepatic steatosis, and other markers of metabolic dysfunction [63]. Besides the WAT and BAT, beige/brite adipose tissue shows intermediate characteristics between white and brown fat, with a central nuclei, multilocular lipid droplets, and is rich in mitochondria [64,65]. In addition to being highly insulin-responsive, adipose tissue also secretes several adipokines involved in glucose regulation and metabolic health [66]. These molecules can act as endocrine regulators, influencing different tissues and organs and regulating local signaling in a paracrine or autocrine manner [67,68]. Beige adipocytes are formed by a process named “WAT browning”, following stimulation of sympathetic activity during chronic cold exposure or administration of β3-adrenergic receptor agonists or exercise [69]. Several pharmacological and nonpharmacological strategies have consequently been developed to induce WAT browning as a possible mechanism to control weight gain and obesity [70]. In fact, adipose organ dysfunction can lead to age-related metabolic alterations, resulting in increased production of inflammatory peptides and macrophage infiltration, and a decrease in anti-inflammatory activity [71]. The production of pro-inflammatory mediators and the infiltration of immune cells inside the tissue generate a state of chronic inflammation. This inflammatory state that worsens with age is defined as “inflammaging” [72]. With aging, there is an increase in the number of white adipocytes and a decreased activity of brown adipocytes. Aging is also associated with a decrease in the formation of beige adipocytes [73]. During obesity, if compared to WAT, brown and beige adipose tissue are less likely to undergo local inflammation, even though an increased production of pro-inflammatory cytokines, such as TNF-alpha and MCP-1 [74], can be observed. However, the inflammation that is generated can compromise the thermogenic activity in BAT due to impaired insulin sensitivity and reduced glucose absorption [75].

## 4. Obesity and Obesity-Related Metabolic Syndrome

Obesity is an abnormal or excessive increase in body fat, classified according to the determination of Body Mass Index (BMI) [76]. According to the World Health Organization (WHO), a BMI greater than or equal to 30 kg/m^2^ is consistent with obesity, which is divided into different grades according to severity, and a BMI between 25 and 30 kg/m^2^ identifies overweight [77]. Excess body fat, especially visceral, is also related to a major risk of numerous chronic diseases including cardiovascular disease, type 2 diabetes, hepatic steatosis, several types of malignancies, and muscular and osteoarticular disorders [78,79]. These diseases are responsible for nearly 3 million deaths per year worldwide. Obesity is the fifth leading cause of death after hypertension, smoking, hyperglycemia, and physical inactivity. A real epidemic of overweight and obese individuals can be detected, especially in undeveloped countries [80,81]. The WHO has predicted a global “obesity epidemic” by 2030, in which 1 in 5 women and 1 in 7 men will be living with obesity. Moreover, this overweight/obesity epidemic and all its complications, have such negative implications on public health, that it can be considered a pandemic disease [82]. Several studies are still underway to better understand the causal factors of obesity. It is a complex hereditary disease whose pathophysiology seems dependent on the interaction between genetics, epigenetics, metagenomics, and environment factors [83,84]. In addition, since the early 1980s, various environmental changes have fostered an “obesogenic environment” with an abundance of high-calorie food, poor-quality food, and, not least, a sedentary lifestyle with reduced physical activity [85]. A long-term imbalance between energy intake and energy expenditure alters the metabolism and functions of WAT. During over-nutrition, lipids are stored within adipocytes [86]. When an enlarged hypertrophic adipocyte reaches maximum capacity, it is no longer able to store excess lipids and becomes fibrotic and inflammatory. Obesity disrupts physiological homeostasis and alters microenvironments by altering stem cell plasticity and impairing regenerative capacity [87,88]. The decreased plasticity of ADSCs exposed to the obese environment could significantly limit their therapeutic potential and ultimately reduce their therapeutic efficacy [89,90]. Excessive adiposity leads to hyperplasia of adipocytes and the secretion of growth factors, such as insulin-like growth factor-1 (IGF-1), tumor necrosis factor-α (TNF-α), angiotensin II (Ang II), and macrophage colony-stimulating factor (M-CSF) [91,92]. Macrophages and other immune cells produce pro-inflammatory cytokines and reactive oxygen species (ROS) that contribute to the development of a state of chronic low-grade inflammation and insulin resistance [93]. In addition, specific adipokines secreted by adipocytes increase vasomotor endothelial tone and consequently hypertension in obese patients [94]. Obesity can lead to increased synthesis and secretion of low-density lipoprotein (LDL) and very low-density lipoprotein (VLDL), which in turn release triglycerides into extrahepatic tissues. High plasma levels of free fatty acids (FFA) inhibit lipogenesis, preventing proper clearance of serum triacylglycerol levels, leading to hypertriglyceridemia and may result in insulin receptor dysfunction [95,96]. Epigenetic changes are crucial for several key biological processes, including cellular differentiation. Recent evidence suggests that obesity may result from the complex interplay between environmental changes and the epigenome [97,98,99]. Indeed, many genes are activated or inhibited to regulate energy metabolism. For example, PPARγ promoter methylation is increased in the subcutaneous adipose tissue of obese women [100]. DNMT1-deficient mice exhibit reduced energy expenditure, increased body weight, and susceptibility to diet-induced obesity [101]. HDAC1 deletion has been found to significantly increase the expression of the thermogenic genes UCP1 and Peroxisome proliferator-activated receptor-gamma coactivator (PGC-1α) by increasing acetylation and decreasing methylation of histone H3 lysine 27 (H3K27) [102]. Overexpression of HDAC4 in adipocytes leads to the expansion of beige adipocytes and a reduction in adiposity [103]. Stem cell or brown adipose tissue transplantation, cell lysates, and exosomes have been tested in obese mouse models [104,105]. Overall, ADSCs were found to be effective in treating obesity-associated diabetes and inflammation and protective against cardiovascular disease [106,107]. Therefore, stem cell therapy represents a promising treatment strategy for obesity and obesity-related comorbidities [108].

## 5. Surgical Management of Obesity

**Figure 2:**
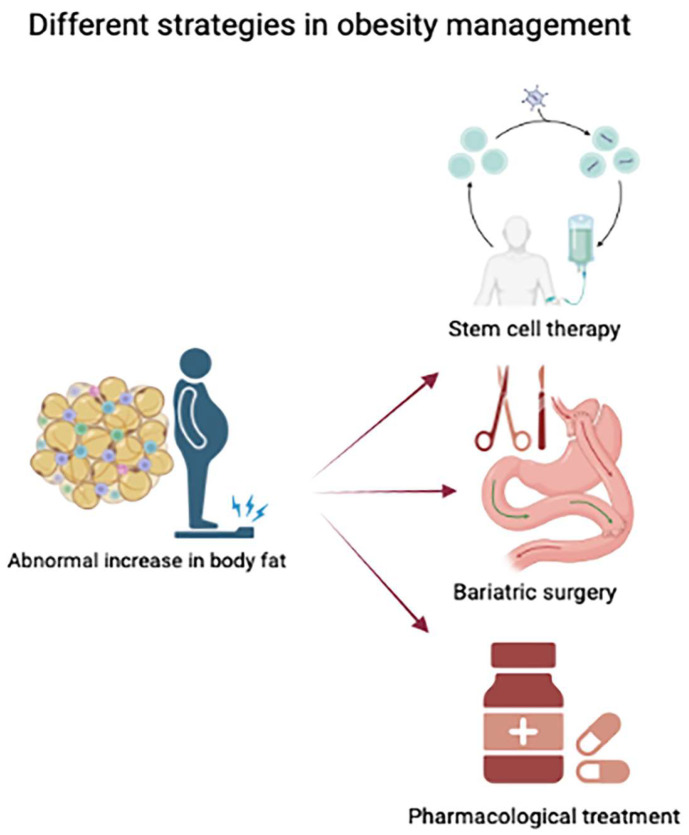
Some therapeutical approaches for management of obesity. Created with BioRender.com accessed on 30 December 2022.

Obesity represents an emerging worldwide disease, with an increased incidence in younger people, that has prompted the development of new and even more effective therapies [109]. Bariatric surgery, also called weight loss surgery, represents one of the approaches for the treatment of severe obesity, with the development of less invasive methods than in the past (Figure 2) [110,111]. Bariatric surgery procedures work by modifying the digestive system, trying to prevent many metabolic obesity-related diseases. The history of obesity surgery is studded with surgical techniques [112]. However, a few main groups can be identified: (1) pure malabsorption (jejunum-ileal bypass or biliointestinal bypass); (2) restrictive (gastroplasties and gastric banding); (3) mixed (bilio-pancreatic diversion and gastric bypass); and (4) alternatives (intragastric balloon and gastric stimulator) [113]. These kinds of procedures are indicated in patients with BMI >40 kg/m^2^ or >35 kg/m^2^ with complications associated with obesity, and in those who do not improve with medical therapy [114,115]. The gastric sleeve, also called sleeve gastrectomy, is one of the most performed bariatric surgeries [116]. It is a simple procedure that removes a large portion of the stomach, leaving behind a small, tubular portion, like a sleeve. This reduces the intake of food and the secretion of hunger hormones [117]. Gastric bypasses modify the stomach, making it Y-shaped by creating a small pouch in the upper part of the stomach [118]. Food flows through the new smaller stomach and the lower segment of the small intestine, bypassing the rest. The small intestine restriction makes this method more effective than gastric restriction alone [119]. Biliopancreatic diversion with duodenal switch (BPD-DS) is similar to gastric bypass but more extreme [120]. This surgery bypasses most of the small intestine, significantly reducing the hunger hormones produced in the small intestine and stomach, and greatly limiting the amount of nutrition the small intestine can absorb [121]. Stomach Intestinal Pylorus Sparing Surgery (SIPS) is a modified version of the original duodenal switch [122]. It begins with a sleeve gastrectomy, and divides the first part of the small intestine by closing it back into a loop; in this type of surgery, a smaller part of the small intestine is bypassed, allowing for greater nutrient absorption [123]. These kinds of procedures involve several complications that occur in the short and long term. Early complications include thromboembolism, pulmonary or respiratory failure, hemorrhage, peritonitis, and wound infection [124]. Late complications include gastrointestinal obstruction, marginal ulceration, band malfunction, steatorrhea, diarrhea, micronutrient nutritional deficiencies, and neurological complications [125,126]. This also occurs in general surgery. Excess subcutaneous adipose tissue leads to impaired healing due to low regional perfusion and oxygen tension. Second, the operative time for the obese is quite long and is a significant predictor of postoperative wound infections [127]. The new style of thinking aims to develop less invasive methods in approaching the obese surgical patients [128]. Almost all bariatric surgeons follow a sequence of application of the various techniques available, in which these are ordered according to the criteria of increasing invasiveness but at the same time effectiveness [129,130]. The beginning of this course is represented by the endoscopic placement of an endogastric board (BIB), which is followed in almost all patients by restrictive surgery [131]. This intervention is represented in many cases by adjustable gastric banding (LAGB), which has the advantage of being completely reversible, as it does not involve mutilation of any part of the digestive tract or anastomosis, and of being easily performed laparoscopically [132,133]. Laparoscopic procedures are associated with shorter operative time, less postoperative pain, and earlier recovery, as well as better respiratory function and aesthetic results [134]. The Swedish Obese Subjects (SOS) study showed that weight loss achieved after bariatric surgery significantly improves all obesity-related risk factors such as diabetes, hypercholesterolemia, low lipoprotein levels, hypertension, and hyperuricemia [135,136]. Weight loss after bariatric surgery is also associated with a significant reduction in overall mortality [137].

## 6. Pharmacological Interventions for Obesity

Pharmacological treatment of obesity became a hot topic in the scientific community (Figure 2), due to the possible wide number of subjects that could potentially benefit, also because behavioral approaches commonly failed and indications for bariatric surgery are becoming narrower [138]. Drugs used to treat obesity can be divided accordingly to their mechanisms of action [139].

### 6.1. Sympathicomimetic

These compounds are generally used for a short-term treatment of obesity. A weight regain after their discontinuation has been also reported [140]. Among these drugs, two compounds are the most used in the clinical practice: diethylpropion, is an amphetamine derivate and a sympathomimetic stimulant which stimulates the endogenous release of dopamine and norepinephrine causing a suppression of appetite. Further, diethylpropion indirectly elevated leptin level in the brain, thus inhibiting the production of neuropeptide Y [141]. The second compound is phentermine that, likewise to diethylpropion, acts through appetite suppression and by increasing basal energy expenditure. Approved by Food and Drug Administration for short-term use (3 months), a recent trial reported an 8.1 Kg loss after 12 weeks of clinical trial with phentermine, as compared to the placebo [142]. Due to the presence of specific side effects (irritability, mood changes, insomnia, elevation in mean blood pressure and palpitations), its use is discouraged in patients with cardiovascular disease, hyperthyroidism, and anxiety. 

### 6.2. Sympathicomimetic and Anticonvulsivant

Phentermine–topiramate: this combined therapy was approved by FDA in 2012 for the chronic treatment of weight control. The appetite suppressant property of phentermine has been associated with the anti-convulsing drug topiramate which showed weight loss potential. Indeed, it has been hypothesized that topiramate has an appetite suppressant effect by modulating GABA receptor activation [143]. The recommend dose for this combined therapy is 7.5 mg/46 mg every day and was shown to induce a mean weight loss of 9.6 Kg after 108 weeks of treatment. The use of higher dosage (15 mg/92 mg) was associated with a further reduction in body weight [144].

### 6.3. Pancreatic Lipase Inhibitor 

Orlistat was approved both by FDA and European Medicines Agency (EMA) for chronic weight management. Orlistat inhibits pancreas and stomach lipase, thus causing a decrease in fat absorption and a reduction in caloric intake [145]. Common side effects have been reported to be diarrhea, flatulence, and abdominal pains, as well as fat-soluble vitamin deficiencies [146] and scanty cases of liver-injuries [147].

### 6.4. 5-HT_2c_ Serotonin Agonist

Lorcaserin is a serotonin receptor agonist with high affinity for 5-HT2C receptor, thus limiting the onset of hallucinations and side effects at the cardiac level, respectively caused by the binding of 5-HT_2A_ and 5-HT_2B_ receptors [148]. Despite the possibility of additional side effect (such as headache, nausea, dry mouth, dizziness, and constipation), the use of lorcaserin at 10 mg twice a day was shown to decrease the body weight by at least 5% in 47.5% of the patients, while 22.6% of them had a weight loss ≥ 10% after 1 year of follow-up [149].

### 6.5. Glucagon-like Peptide 1 Agonists

Liraglutide is a glucagon-like peptide 1 (GLP-1) agonist, approved by both FDA and EMA for chronic weight management. GLP-1 is physiologically secreted by the enteroendocrine L-cells of distal ileum and proximal colon after oral meal consumption, but a certain basal rate of secretion has been recently postulated [150]. GLP-1 is an incretin hormone which increases the glucose-dependent insulin secretion but has shown additional effects: decreases in appetite and food consumption [151], and delays in gastric emptying, thus increasing postprandial feelings of satiety and fullness [152]. The use of Liraglutide was associated with a mean reduction of 8.4 Kg in body weight after 1 year of follow-up and a reduction in cardiovascular risk factors [153]. Gastrointestinal side effects have been reported in some studies [154].

### 6.6. Opioid Receptor Antagonist/Dopamine and Noradrenaline Reuptake Inhibitor 

Naltrexone–bupropion: naltrexone is an opioid antagonist prescribed for the management of alcohol and opioid use disorder. Bupropion is a dopamine and norepinephrine neuronal reuptake inhibitor and was firstly prescribed as an antidepressant. Bupropion also stimulates the α-MSH release, thus inducing appetite regulation. While the exact underlying mechanism is still unknown, it has been suggested that naltrexone and bupropion synergistically act at the melanocortin system [155]. Their combined use is approved by EMA and FDA and has been associated with a mean reduction of 6.1% in body weight [156].

## 7. Conclusions

Dysfunction of adipocytes and adipose tissue is the main feature of obesity, resulting in an increased risk of insulin resistance, type 2 diabetes, fatty liver disease, hypertension, dyslipidemia, and cardiovascular disorders. In most obese subjects, the compromised physiology of the adipose tissue depends on the hypertrophy of the adipocytes and on the interaction of genetic, epigenetic, and environmental factors. Several surgical procedures, such as biliointestinal bypass or gastric banding, and pharmacological interventions, such as pancreatic lipase inhibitors or Glucagon-Like Peptide 1 agonists, are applied for the management of obesity. Therefore, stem cell therapy and the use of targeted treatment acting on cell differentiation or epigenetic modifications of key target genes, may represent a promising strategy for the management of obesity and obesity-related metabolic syndrome.

## Data Availability

Not applicable.
